# Hand-assisted laparoscopic surgery for an esophageal hiatal hernia with incarcerated transverse colon presenting after laparoscopic gastrectomy: a case report

**DOI:** 10.1186/s40792-023-01621-y

**Published:** 2023-03-20

**Authors:** Shingo Itamoto, Nobuaki Fujikuni, Kazuaki Tanabe, Senichiro Yanagawa, Masahiro Nakahara, Toshio Noriyuki

**Affiliations:** 1 Department of Surgery, Onomichi General Hospital, 1-10-23 Hirahara, Onomichi, Hiroshima 722-8508 Japan; 2 Department of Gastroenterological Surgery, Hiroshima Prefectural Hospital, 1-5-54 Ujinakanda, Minami, Hiroshima, Hiroshima 734-8530 Japan; 3 Department of Perioperative and Critical Care Management, Graduate School of Biomedical and Health Sciences, Hiroshima University, 1-2-3 Kasumi, Minami, Hiroshima, Hiroshima 734-8551 Japan

**Keywords:** Esophageal hiatal hernia, EHH, Hand-assisted laparoscopic surgery, HALS

## Abstract

**Background:**

Esophageal hiatal hernia (EHH) presenting after gastrectomy for carcinoma is a type of internal hernia and very rare. There have been no published reports on the use of hand-assisted laparoscopic surgery (HALS) for the treatment of an incarcerated EHH that presented after a gastrectomy. Herein, we report a rare case of HALS performed for an incarcerated EHH presenting after a laparoscopic gastrectomy.

**Case presentation:**

This case report presents the case of a 66-year-old man who underwent hernia repair for an incarcerated hernia that presented after he underwent a laparoscopic proximal gastrectomy with double-tract reconstruction for cancer in the esophagogastric junction. Emergency laparoscopic hernia repair was performed and herniation of the transverse colon into the left thoracic cavity through a hiatal defect was confirmed. Since it was difficult to return the transverse colon into the abdominal cavity using forceps, the procedure was converted to HALS and the transverse colon was pulled back into the abdominal cavity. The hernia defect was closed using a non-absorbable suture. The postoperative course was uneventful, and the patient was discharged on the fourth postoperative day.

**Conclusions:**

The HALS approach provides the tactile experience of an open surgery combined with the benefits of a laparoscopic procedure such as good visualization and low invasiveness. In this case, when the transverse colon that had herniated into the left hemithorax was returned to the abdominal cavity, damage to the transverse colon was avoided by using the hand. Hence, HALS was safely performed to repair an incarcerated EHH after gastrectomy.

## Background

Postoperative internal herniation is the protrusion of the viscus through the mesenteric or peritoneal aperture after a surgery [1]. Esophageal hiatal hernia (EHH) is a type of internal hernia. The incidence of symptomatic EHH following esophageal and gastric resection for carcinoma is 2.8%, while the median time between the primary surgery and the diagnosis of an EHH is 15 months [2].

EHH presenting after gastrectomy is divided into three types: (1) conventional, which includes herniated contents other than the alimentary limb; (2) migration, which involves mediastinal migration of the esophagojejunostomy through the esophageal hiatus; and (3) both [3]. To our knowledge, only four cases of incarcerated EHH after gastrectomy have been reported in the literature [4–7], and there have been no published reports of the use of hand-assisted laparoscopic surgery (HALS) for an incarcerated EHH presenting after a gastrectomy. Here, we report a rare case of HALS used to treat an incarcerated EHH after laparoscopic gastrectomy.

## Case presentation

**Fig. 1:**
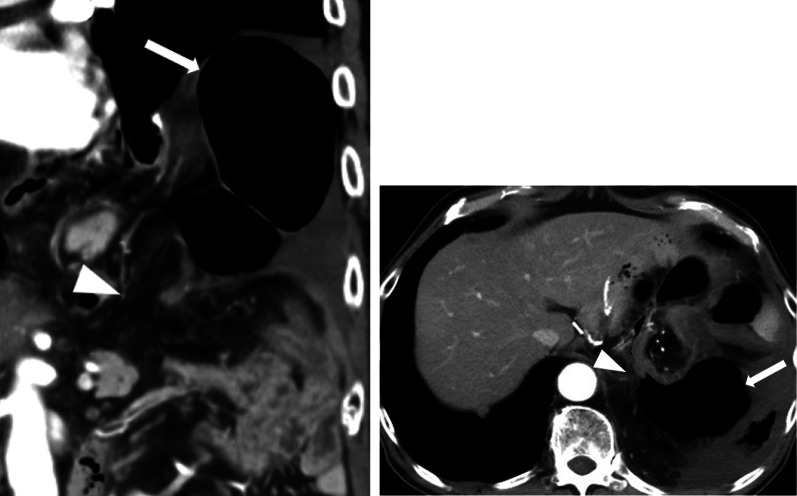
Computed tomography with intravenous contrast demonstrating a transverse colon (arrow) that has herniated into the left hemithorax through the hernial defect (arrowhead)

**Fig. 2:**
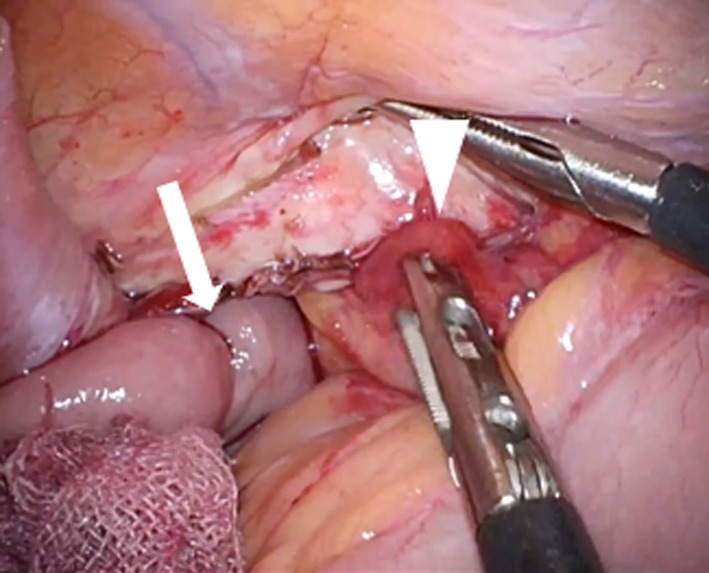
Intraoperative findings reveal herniation of the transverse colon (arrowhead) which is on the left side of elevated jejunum (arrow) into the left hemithorax

**Fig. 3:**
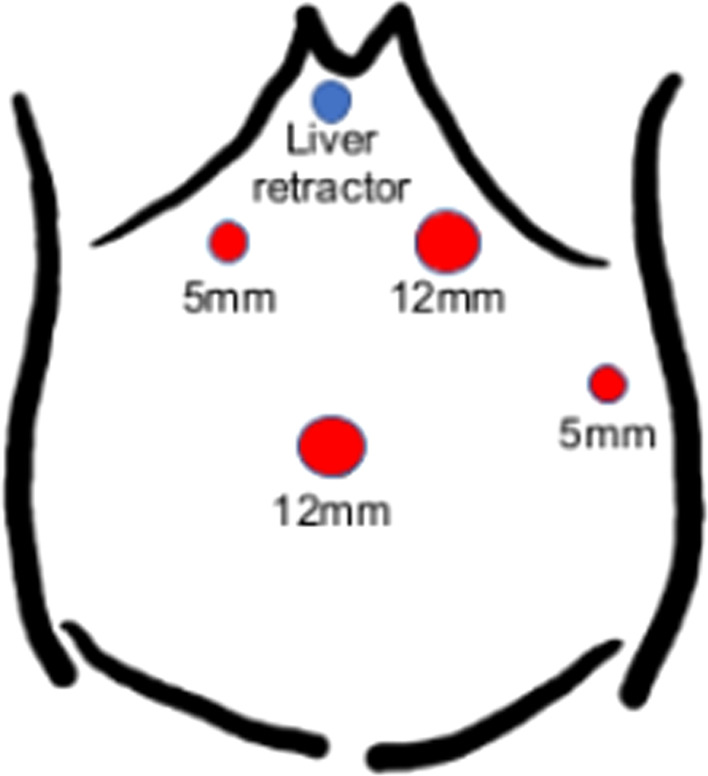
Port arrangement: the skin incision of the liver retractor was extended and a GelPort® attached for use as a hand access site

**Fig. 4:**
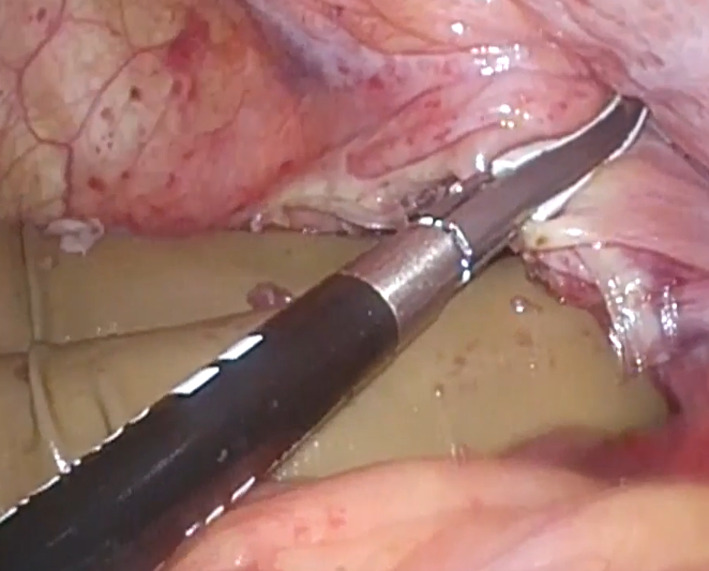
Left hand is inserted behind the diaphragm, which is subsequently cut due to difficulty returning the transverse colon into the abdominal cavity

**Fig. 5:**
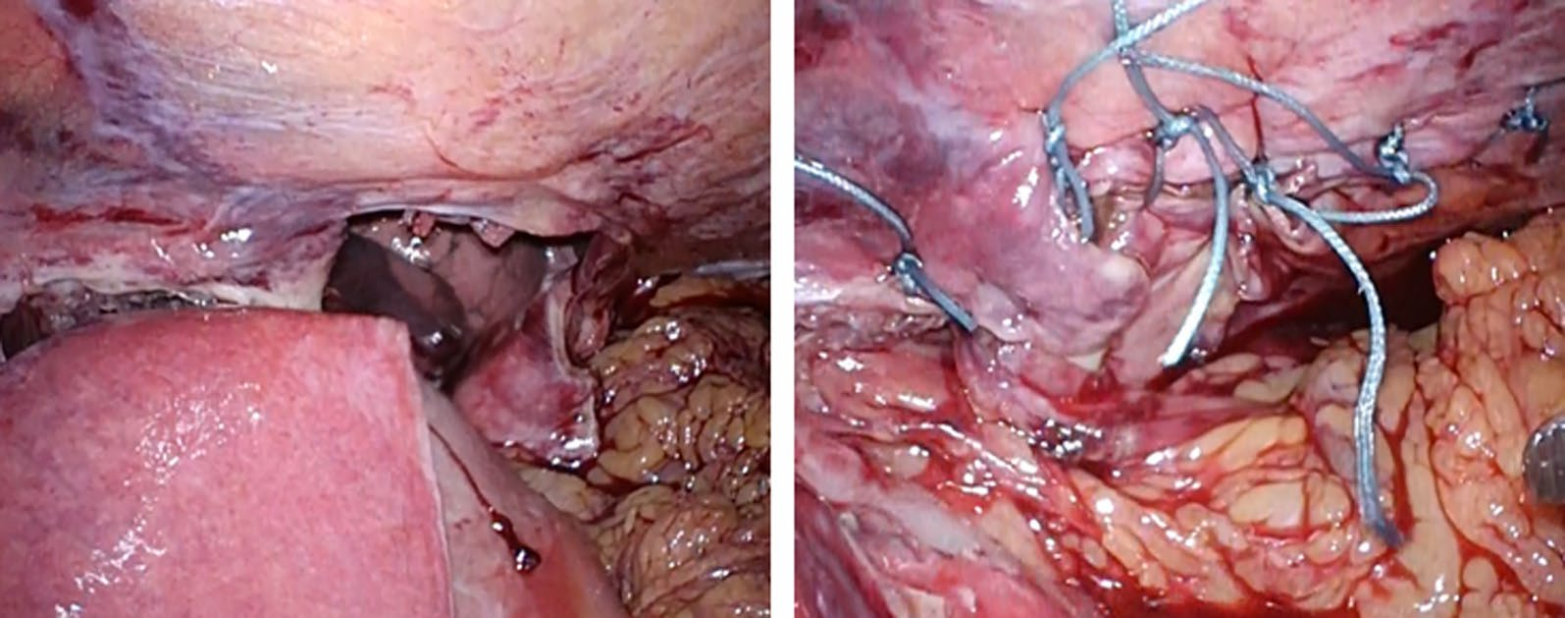
Hernia defect is closed with a non-absorbable suture

A 66-year-old man presented to our emergency department with a sudden acute onset of severe upper left abdominal pain and nausea. He was previously diagnosed with cancer of the esophagogastric junction, classified as T3N1M0 stage IIB based on TNM classification. He underwent laparoscopic proximal gastrectomy and D2 lymphadenectomy with double tract reconstruction 16 months prior to the current presentation. We opened the right and left crus widely to create sufficient space for esophagectomy and anastomosis and cut the left mediastinal pleura to open the left thoracic cavity through the esophageal hiatus. After the reconstruction, we continuously sutured the diaphragm and elevated small intestine using a 3-0 V-Loc® to prevent hiatal hernia. The patient also received postoperative adjuvant chemotherapy. He had no history of any allergies for taking any routine medications. His weight on presentation was 68.5 kg, while his body mass index (BMI) was 22.6 kg/m^2^. He had lost 8.0 kg after the gastrectomy. Computed tomography of the abdomen with intravenous contrast revealed an incarcerated hiatal hernia through a hiatal defect, through which the dilated transverse colon herniated into the left hemithorax (Fig. 1). No free air was visible. After the patient provided informed consent, an emergency laparoscopic hernia repair was performed to treat the EHH and transverse colon incarceration. Herniation of the transverse colon into the left thoracic cavity through a hiatal defect was confirmed (Fig. 2). Since it was difficult to return the transverse colon into the abdominal cavity using forceps, the procedure was converted to HALS (Fig. 3). The diaphragm on the left side of the hernia defect was dissected (Fig. 4). The transverse colon could not be pulled back into the abdominal cavity, because the omentum was adherent to the transverse. Therefore, the omentum was separated from the transverse colon, which was pulled back into the abdominal cavity. No intestinal resection was performed because there were no signs of ischemia. The hernia defect was closed using non-absorbable sutures (Fig. 5). The total operation time was 152 min, and the ascites loss was 600 mL. The intraoperative blood loss was low. The postoperative course was uneventful, and the patient was discharged on the fourth postoperative day.

## Discussion

The incidence of a symptomatic EHH after esophageal and gastric resection for carcinoma is 2.8% (total/subtotal gastrectomy, 0.7%; transthoracic esophagectomy, 2.7%; and extended gastrectomy, 6.1%), while the median time between the primary surgery and the diagnosis of EHH is 15 months [2]. Hence, based on this incidence of symptomatic EHH, the incidence of an incarcerated EHH after a gastrectomy is exceptionally rare. Table 1 summarizes a total of five cases of incarcerated EHH after gastrectomy reported in the literature, including our case [4–7]. Four patients underwent resection of the diaphragmatic crus for esophageal jejunal anastomosis. In addition, three of four patients who underwent resection of the diaphragmatic crus did not undergo crus repair. Tashiro et al. proposed that EHH could be caused by negative intrathoracic pressure, resection of the diaphragmatic crus, low BMI, and liver cirrhosis [8]. Ito et al. suggested that crus incision without repair is associated with EHH after laparoscopic total gastrectomy [3]. They also concluded that reduced division of the crus and dissection of the esophagus are preferable, but if necessary, suture repair may help avoid postoperative EHH [3]. In our patient, resection of the diaphragmatic crus, weight loss after gastrectomy, laparoscopy-assisted gastrectomy and thoracotomy through an esophageal hiatus were considered to have caused the EHH.

**Table 1:** Summary of a total of five cases of incarcerated EHH

No	Author	Years	Age/Sex	Symptom	Primary surgery	Crus incision and repair in primary surgery	Type of anastomosis	Interval	Incarcerated organ	Signs of ischemia	Surgical approach	Surgical procedure	Open convert
1	Murata [4]	2014	44/M	Dyspnea and chest pain	TG with R-Y	Yes/Yes	Esophageal jejunal anastomosis	2 days	Transverse colon	No	Open	Close the hernia defect	–
2	Santos [5]	2016	76/M	Vomiting and abdominal pain	LTG with R-Y	Yes/No	Esophageal jejunal anastomosis	60 days	Small intestine	Yes	Laparoscopy	Resect ischemic lesion with enteric anastomosis and close the hernia defect	No
3	Nai-Yu Wang [6]	2019	76/M	Abdominal fullness	LTG with R-Y	Yes/No	Esophageal jejunal anastomosis	8 days	Jejunal limb	Yes	Open	Resect ischemic lesion with enteric anastomosis and close the hernia defect	–
4	Mohsen Ezzy [7]	2021	66/M	Nausea and abdominal pain	LTG with R-Y	Unknown/unknown	Esophageal jejunal anastomosis	1 year	Small intestine	No	Open	Close the hernia defect	–
5	Our case	2021	66/M	Nausea and abdominal pain	LPG with double tract reconstruction	Yes/No	Esophageal jejunal anastomosis	1.5 years	Transverse colon	No	HALS	Close the hernia defect	No

EHH: esophageal hiatal hernia, M: male, F: female, LTG: laparoscopic total gastrectomy, TG: total gastrectomy, LPG: laparoscopic proximal gastrectomy, R-Y: Roux-en-Y reconstruction, HALS: hand assisted laparoscopic surgery

The most common presentation of an internal hernia is abdominal pain, followed by nausea and vomiting [9]. Making the early diagnosis of a complicated hiatal hernia after gastrectomy is often challenging because of the nonspecific symptoms and a variety of differential diagnoses. Therefore, a high index of suspicion and the use of appropriate diagnostic imaging modalities are paramount [3]. When diagnosed early, only repair of the hernial defect is required, which results in lower morbidity and mortality rates. However, if the diagnosis is delayed, rupture of the small intestine may occur, leading to mediastinitis, which can be life-threatening [5]. If irreversible ischemic necrotic tissue or a perforation is discovered, resection and repair should be performed as necessary. In the present case, the patient presented with abdominal pain and nausea. No intestinal resection was performed because there were no signs of ischemia.

HALS is a hybrid procedure that allows the surgeon to insert their non-dominant hand into the abdomen under laparoscopic guidance. This approach provides the tactile experience of open surgery, combined with the benefits of a laparoscopic procedure, such as good visualization and low invasiveness [10–12]. In the present case, repair of the transverse colon was difficult because of adhesions, a narrow hiatus, and colonic edema. Damage to the invaginated transverse colon can be avoided by insertion of the left hand behind the diaphragm during the dissection. In addition, returning the transverse colon, which had herniated into the left hemithorax, back into the abdominal cavity, was gentler with the hand than with forceps. Based on these findings, HALS should be considered an alternative to laparoscopic surgery for incarcerated EHH repair after gastrectomy.

## Conclusions

In this case, HALS was safely and successfully performed to repair an incarcerated EHH. Since HALS allows gentle manipulation of the intestinal tract, it should be considered a treatment approach for incarcerated EHH after gastrectomy.

## Data Availability

Data sharing is not applicable to this article, as no datasets were generated or analyzed during the current study.

## Abbreviations

EHH: Esophageal hiatal hernia
HALS: Hand assisted laparoscopic surgery
BMI: Body mass index
